# Supplementary material for: Forecasting monthly runoff in a glacierized catchment: A comparison of extreme gradient boosting (XGBoost) and deep learning models
Source: PLoS One. 2025 May 23;20(5):e0321008. doi: 10.1371/journal.pone.0321008 (PMC12101857; doi:10.1371/journal.pone.0321008)
Supplement: S1 Table — (DOCX) [file pone.0321008.s001.docx]

**S Appendix**

Table S1. Monthly runoff data that is used in this study.

| Year | January | February | March | April | May | June | July | August | September | October | November | December |
| --- | --- | --- | --- | --- | --- | --- | --- | --- | --- | --- | --- | --- |
| 2002 | 0.547 | 0.513 | 0.690 | 1.558 | 4.889 | 13.723 | 12.554 | 9.897 | 6.086 | 2.321 | 2.120 | 1.007 |
| 2003 | 0.764 | 0.595 | 0.734 | 1.856 | 6.556 | 16.778 | 13.430 | 14.962 | 5.552 | 2.180 | 0.929 | 0.671 |
| 2004 | 0.578 | 0.507 | 0.560 | 1.310 | 3.817 | 8.473 | 11.731 | 12.386 | 7.891 | 2.927 | 2.374 | 0.850 |
| 2005 | 0.629 | 0.539 | 0.681 | 1.218 | 4.797 | 10.923 | 10.868 | 9.208 | 6.910 | 2.437 | 1.207 | 0.615 |
| 2006 | 0.505 | 0.461 | 0.490 | 1.292 | 6.025 | 12.523 | 15.468 | 6.331 | 7.088 | 3.839 | 1.184 | 0.748 |
| 2007 | 0.686 | 0.590 | 0.645 | 3.466 | 6.191 | 11.588 | 12.628 | 11.960 | 6.151 | 3.757 | 1.082 | 0.659 |
| 2008 | 0.524 | 0.536 | 0.608 | 0.956 | 8.078 | 12.984 | 12.146 | 11.301 | 6.821 | 1.962 | 1.303 | 0.794 |
| 2009 | 0.608 | 0.510 | 0.519 | 1.574 | 7.578 | 10.582 | 11.683 | 13.041 | 7.124 | 3.067 | 1.176 | 0.794 |
| 2010 | 0.640 | 0.526 | 0.622 | 1.838 | 4.344 | 11.658 | 13.573 | 8.913 | 4.523 | 1.984 | 1.059 | 0.733 |
| 2011 | 0.551 | 0.478 | 0.621 | 2.425 | 4.622 | 8.649 | 9.547 | 11.443 | 8.565 | 4.197 | 1.267 | 0.904 |
| 2012 | 0.705 | 0.583 | 0.929 | 1.672 | 6.150 | 13.337 | 14.029 | 12.555 | 5.806 | 3.008 | 1.150 | 0.786 |
| 2013 | 0.624 | 0.555 | 0.513 | 1.830 | 4.560 | 11.013 | 14.061 | 11.035 | 5.656 | 2.825 | 1.310 | 0.800 |
| 2014 | 0.630 | 0.537 | 0.944 | 2.080 | 3.282 | 8.201 | 10.554 | 9.500 | 5.089 | 3.016 | 1.318 | 0.867 |
| 2015 | 0.684 | 0.570 | 0.619 | 1.651 | 6.338 | 10.858 | 14.421 | 11.981 | 5.372 | 1.974 | 1.289 | 0.719 |
| 2016 | 0.488 | 0.549 | 0.503 | 2.154 | 4.572 | 9.875 | 13.073 | 9.941 | 6.996 | 1.724 | 0.968 | 0.738 |
| 2017 | 0.537 | 0.514 | 0.808 | 2.072 | 5.298 | 11.624 | 9.638 | 11.016 | 3.103 | 2.768 | 0.955 | 0.644 |
| 2018 | 0.500 | 0.494 | 0.444 | 2.183 | 8.401 | 12.063 | 11.121 | 10.146 | 7.340 | 2.916 | 1.115 | 0.761 |
| 2019 | 0.593 | 0.563 | 0.722 | 1.622 | 3.016 | 16.887 | 15.175 | 13.752 | 6.513 | 3.364 | 1.638 | 0.941 |
| 2020 | 0.671 | 0.734 | 1.013 | 3.805 | 8.438 | 10.203 | 15.044 | 11.540 | 6.666 | 3.063 | 1.509 | 0.861 |
| 2021 | 0.550 | 0.458 | 0.801 | 1.527 | 3.331 | 16.445 | 16.343 | 11.611 | 7.083 | 2.192 | 0.963 | 0.700 |

Table S2. Hyperparameters for the applied models

| Models | Parameters |
| --- | --- |
| LSTM | Hidden unites = 25 |
|  | Activation for hidden layer is Rectified Linear Unit (relu) |
|  | Dropout = 0.12 |
|  | Activation for Dense layer (output layer) is sigmoid |
|  | Learning rate=0.001 |
|  | Optimizer is Adam |
|  | Epochs = 300 |
|  | Batch size = 20 |
| RF | Number of trees in the forest = 20 |
|  | Seed for reproducibility = 18 |
|  | Minimum number of samples required to be at a leaf node = 5 |
|  | Minimum number of samples required to split an internal node = 3 |
| XGBoost | Learning rate = 0.15 |
|  | Maximum depth of a tree = 7 |
|  | Subsample ratio of the training instance = 0.19 |
|  | subsample ratio of columns when constructing each tree = 0.75 |
|  | minimum loss reduction = 0.08 |
|  | L2 regularization term on weights = 1 |
|  | L1 regularization term on weights = 0.09 |
|  | Minimum sum of instance weight needed in a child =2 |
