# Supplementary material for: Forecasting monthly runoff in a glacierized catchment: A comparison of extreme gradient boosting (XGBoost) and deep learning models
Source: PLoS One. 2025 May 23;20(5):e0321008. doi: 10.1371/journal.pone.0321008 (PMC12101857; doi:10.1371/journal.pone.0321008)
Supplement: Attachment — Submitted filename: Response to Reviewers.docx [file pone.0321008.s004.docx]

**Dear Editor,**

We sincerely appreciate the opportunity to submit the revised version of our manuscript entitled “**Forecasting Monthly Runoff in a Glacierized Catchment: A Comparison of Extreme Gradient Boosting (XGBoost) and Deep Learning Models.**” to PLOS ONE.

We would also like to express our gratitude to the esteemed reviewers for their valuable and constructive comments. We have worked diligently to ensure that all authors have collaboratively addressed the comments raised by the reviewers and have made the necessary revisions to enhance the manuscript. Additionally, we would like to confirm that we have responded to the editorial comments, including the copyright concerns regarding Figure 1. We utilized a map sourced from open access source (please see Figure 1 in the main paper), and the relevant data availability details are incorporated within the manuscript and provided in the Appendix (please see the Suplimantary_file). Furthermore, our manuscript specifies that the models were developed using Python, which is open-access and freely available.

**Below, we provide a point-by-point response to the comments raised by the reviewers:**

Reviewer #1:

In this work, the Extreme Gradient Boosting (XGBoost), random forest (RF) and long-short term memory (LSTM) model was used to forecast monthly runoff for one month ahead of time in the Lotschental watershed in Switzerland. The work is well organized. however, with the following minor corrections, it is acceptable:

SPECIFIC RECOMMENDATIONS
ABSTRACT
- The abstract could briefly explain why these methods were chosen or how they contribute to the research.

*Reply: We have explained in the abstract the reasons for selecting the applied models, as per your suggestion.*

- The results could be more specific, including quantitative outcomes or more detailed.

*Reply: We have incorporated additional quantitative results and details to make our findings more specific, as suggested.*

-  At the end of the Abstract, it is necessary to point out the global importance of this study for society. This would arouse greater interest from Journal readers.

R*eply: We have emphasized the global significance of this study for society, as recommended.*

INTRODUCTION SECTION
• The introduction effectively covers the necessary elements but could be improved by providing a more detailed summary of methods, emphasizing the scientific gap, and enhancing the problem statement with a broader context.

*Reply: We have enhanced our introduction by including a summary of the applied models and highlighting the existing scientific gaps, as recommended. Please see pages 5 (lines 102 to 110) and 6-7 (lines 133 to 146).*

• If possible, reinforce the introduction with up-to-date references (2022-2024) below. Because it is important to include the most recent articles.

1. Improving the accuracy of rainfall-runoff relationship estimation using signal processing techniques, bio-inspired swarm intelligence and artificial intelligence algorithms
2. Monthly streamflow prediction in Amasya, Türkiye, using an integrated approach of a feedforward backpropagation neural network and discrete wavelet transform
3. Improving the accuracy of rainfall-runoff relationship estimation using signal processing techniques, bio-inspired swarm intelligence and artificial intelligence algorithms
4. Application of empirical mode decomposition, particle swarm optimization, and support vector machine methods to predict stream flows
5. Simulation of the potential impacts of projected climate and land use change on runoff under CMIP6 scenarios

*Reply: We would like to confirm that we have improved our paper by incorporating new references, as suggested. Below is a list of some additional references included in the paper:*

*1.	Al-Kubaisi MHD. Surface Runoff Estimation in Kubaisa Watershed Using SWAT, Western Desert, Iraq. Iraqi Geological Journal. 2024;57: 286–297. doi:10.46717/igj.57.2B.19ms-2024-8-29*

*2.	Javan K, Lialestani MRFH, Nejadhossein M. A comparison of ANN and HSPF models for runoff simulation in Gharehsoo River watershed, Iran. Model Earth Syst Environ. 2015;1: 41. doi:10.1007/s40808-015-0042-1*

*3.	Vishwakarma DK, Kumar P, Yadav KK, Ali R, Markuna S, Chauhan S, et al. Evaluation of CatBoost Method for Predicting Weekly Pan Evaporation in Subtropical and Sub-Humid Regions. Pure Appl Geophys. 2024;181: 719–747. doi:10.1007/s00024-023-03426-4*

*4.	Elbeltagi A, Al-Mukhtar M, Kushwaha NL, Al-Ansari N, Vishwakarma DK. Forecasting monthly pan evaporation using hybrid additive regression and data-driven models in a semi-arid environment. Appl Water Sci. 2022;13: 42. doi:10.1007/s13201-022-01846-6*

*5.	Amiri E. Forecasting daily river flows using nonlinear time series models. J Hydrol (Amst). 2015;527: 1054–1072. doi:https://doi.org/10.1016/j.jhydrol.2015.05.048*

*6.	Wang S, Peng H. Multiple spatio-temporal scale runoff forecasting and driving mechanism exploration by K-means optimized XGBoost and SHAP. J Hydrol (Amst). 2024;630: 130650. doi:https://doi.org/10.1016/j.jhydrol.2024.130650*

*7.	Guo J, Liu Y, Zou Q, Ye L, Zhu S, Zhang H. Study on optimization and combination strategy of multiple daily runoff prediction models coupled with physical mechanism and LSTM. J Hydrol (Amst). 2023;624: 129969. doi:https://doi.org/10.1016/j.jhydrol.2023.129969*

*8.	Xu Y, Lin K, Hu C, Wang S, Wu Q, Zhang J, et al. Interpretable machine learning on large samples for supporting runoff estimation in ungauged basins. J Hydrol (Amst). 2024;639: 131598. doi:https://doi.org/10.1016/j.jhydrol.2024.131598*

*9.	Wang Y, Wang W, Xu D, Zhao Y, Zang H. A compound approach for ten-day runoff prediction by coupling wavelet denoising, attention mechanism, and LSTM based on GPU parallel acceleration technology. Earth Sci Inform. 2024;17: 1281–1299. doi:10.1007/s12145-023-01212-3*

*10.	Guo H, Chen L, Fang Y, Zhang S. Model and application of annual river runoff prediction based on complementary set empirical mode decomposition combined with particle swarm optimization adaptive neuro-fuzzy system. Water Supply. 2023;23: 1760–1774. doi:10.2166/ws.2023.075*

*11.	Kumar K, Singh V, Roshni T. Application of the PSO–neural network in rainfall–runoff modeling. Water Pract Technol. 2022;18: 16–26. doi:10.2166/wpt.2022.155*

*12.	Katipoğlu OM, Sarıgöl M. Improving the accuracy of rainfall-runoff relationship estimation using signal processing techniques, bio-inspired swarm intelligence and artificial intelligence algorithms. Earth Sci Inform. 2023;16: 3125–3141. doi:10.1007/s12145-023-01081-w*

*13.	Katipoğlu OM, Yeşilyurt SN, Dalkılıç HY, Akar F. Application of empirical mode decomposition, particle swarm optimization, and support vector machine methods to predict stream flows. Environ Monit Assess. 2023;195: 1108. doi:10.1007/s10661-023-11700-0*

*14.	Hameed MM, Mohd Razali SF, Wan Mohtar WHM, Yaseen ZM. Examining optimized machine learning models for accurate multi-month drought forecasting: A representative case study in the USA. Environmental Science and Pollution Research. 2024. doi:10.1007/s11356-024-34500-6*

*15. Hameed MM, Masood A, Srivastava A, Abd Rahman N, Mohd Razali SF, Salem A, et al. Investigating a hybrid extreme learning machine coupled with Dingo Optimization Algorithm for modeling liquefaction triggering in sand-silt mixtures. Sci Rep. 2024;14: 10799. doi:10.1038/s41598-024-61059-6*

*15. Alomar MK, Khaleel F, Aljumaily MM, Masood A, Razali SFM, AlSaadi MA, et al. Data-driven models for atmospheric air temperature forecasting at a continental climate region. PLOS ONE. 2022;17: e0277079.*

*16.	Döll P, Abbasi M, Messager ML, Trautmann T, Lehner B, Lamouroux N. Streamflow Intermittence in Europe: Estimating High-Resolution Monthly Time Series by Downscaling of Simulated Runoff and Random Forest Modeling. Water Resources Research. 2024;60: e2023WR036900. doi:https://doi.org/10.1029/2023WR036900*

*17.	Zema DA, Parhizkar M, Plaza-Alvarez PA, Xu X, Lucas-Borja ME. Using random forest and multiple-regression models to predict changes in surface runoff and soil erosion after prescribed fire. Modeling Earth Systems and Environment. 2024;10: 1215–1228. doi:10.1007/s40808-023-01838-8*

MATERIALS & METHOD
METHODOLOGY
• Consider adding brief explanations on why specific methods were chosen.

*Reply: We provide a brief explanation of the main reasons for using these models in runoff forecasting. Please refer to page 13 (lines 246 to 258) for more details.*

RESULTS
• Improve the logical flow and ensure that each finding is clearly explained.

*Reply: We have improved the logical flow of the results section as suggested and have clearly presented the findings of this research. Please refer to pages 23, 26, 27, and 28, for details.*

•Please give applied model parameters.

*Reply: The model parameters are provided in the Appendix; please see Table S2.*

CONCLUSIONS

• Clearly stating the most significant findings, with specific reference to the data.

• Offer more detailed and actionable recommendations based on the study's results.

• Highlight the study's contribution to existing research and its potential implications for theory and practice.

• Ensure that each paragraph is concise and directly contributes to the discussion.

*Reply: We have revised the Conclusion section in accordance with your constructive comments:*

- *We have clearly explained the key findings, incorporating specific data as suggested.*
- *The major contributions of the study are stated as you recommended.*
- *We have highlighted the potential implications and practical applications of the research findings.*
- *Each paragraph of the Conclusion has been made concise and now directly contributes to the discussion, as suggested.*
- *Recommendations have been provided as advised.*

Reviewer #2:

This paper proposes Extreme Gradient Boosting (XGBoost) and Deep Learning Models to predict the Monthly Runoff in a Glacierized Catchment. However, the quality of the paper should be improved a bit. Specific comments are as follows.

1. The graphical abstract/flow diagram is missing, it must be added.

*Reply: A flow diagram has been added and explained. Please see Figure 5 on page 19.*
2. The novelty statement and research gap should be highlights in innovative aspect of this study, specifically in abstract and Introduction.

*Reply:* *The novelty of the current research has been highlighted in both the introduction and abstract sections, as suggested. Please see page 2 (Abstract) , and pages 6, and 7 (lines 133 – 146), in the introduction section.*
3. The shortcomings of existing research should be summarized, and the innovation of this paper should be highlighted.

*Reply: We have summarized the most essential shortcomings of existing research and highlighted the significance and innovation of our paper in the Introduction section. Please refer to pages 4 (lines, 75-85) and 6, and 7 (lines 133-146, 154-159).*

4. As described in the paper, Extreme Gradient Boosting (XGBoost) and Deep Learning Models has been widely applied in the various fields, such as river stage forecasting (<https://doi.org/10.1007/s13201-024-02103-8>;

<https://doi.org/10.1016/j.heliyon.2023.e16290>), Drought Forecasting (<https://doi.org/10.3390/w15040765>) and long-term rainfall prediction (<https://doi.org/10.1007/s00024-022-03189-4>), Pan Evaporation (<https://doi.org/10.1007/s00024-023-03426-4>; <https://doi.org/10.1007/s13201-022-01846-6>). However, several aspects in health monitoring, such as the reason for the adoption of Extreme Gradient Boosting (XGBoost) and Deep Learning Models in this paper is not clearly explained, it need to be appropriately supplemented.

*Reply: We have improved our paper accordingly. Below is a list of some additional references included in the paper:*

*1.	Al-Kubaisi MHD. Surface Runoff Estimation in Kubaisa Watershed Using SWAT, Western Desert, Iraq. Iraqi Geological Journal. 2024;57: 286–297. doi:10.46717/igj.57.2B.19ms-2024-8-29*

*2.	Javan K, Lialestani MRFH, Nejadhossein M. A comparison of ANN and HSPF models for runoff simulation in Gharehsoo River watershed, Iran. Model Earth Syst Environ. 2015;1: 41. doi:10.1007/s40808-015-0042-1*

*3.	Vishwakarma DK, Kumar P, Yadav KK, Ali R, Markuna S, Chauhan S, et al. Evaluation of CatBoost Method for Predicting Weekly Pan Evaporation in Subtropical and Sub-Humid Regions. Pure Appl Geophys. 2024;181: 719–747. doi:10.1007/s00024-023-03426-4*

*4.	Elbeltagi A, Al-Mukhtar M, Kushwaha NL, Al-Ansari N, Vishwakarma DK. Forecasting monthly pan evaporation using hybrid additive regression and data-driven models in a semi-arid environment. Appl Water Sci. 2022;13: 42. doi:10.1007/s13201-022-01846-6*

*5.	Amiri E. Forecasting daily river flows using nonlinear time series models. J Hydrol (Amst). 2015;527: 1054–1072. doi:https://doi.org/10.1016/j.jhydrol.2015.05.048*

*6.	Wang S, Peng H. Multiple spatio-temporal scale runoff forecasting and driving mechanism exploration by K-means optimized XGBoost and SHAP. J Hydrol (Amst). 2024;630: 130650. doi:https://doi.org/10.1016/j.jhydrol.2024.130650*

*7.	Guo J, Liu Y, Zou Q, Ye L, Zhu S, Zhang H. Study on optimization and combination strategy of multiple daily runoff prediction models coupled with physical mechanism and LSTM. J Hydrol (Amst). 2023;624: 129969. doi:https://doi.org/10.1016/j.jhydrol.2023.129969*

*8.	Xu Y, Lin K, Hu C, Wang S, Wu Q, Zhang J, et al. Interpretable machine learning on large samples for supporting runoff estimation in ungauged basins. J Hydrol (Amst). 2024;639: 131598. doi:https://doi.org/10.1016/j.jhydrol.2024.131598*

*9.	Wang Y, Wang W, Xu D, Zhao Y, Zang H. A compound approach for ten-day runoff prediction by coupling wavelet denoising, attention mechanism, and LSTM based on GPU parallel acceleration technology. Earth Sci Inform. 2024;17: 1281–1299. doi:10.1007/s12145-023-01212-3*

*10.	Guo H, Chen L, Fang Y, Zhang S. Model and application of annual river runoff prediction based on complementary set empirical mode decomposition combined with particle swarm optimization adaptive neuro-fuzzy system. Water Supply. 2023;23: 1760–1774. doi:10.2166/ws.2023.075*

*11.	Kumar K, Singh V, Roshni T. Application of the PSO–neural network in rainfall–runoff modeling. Water Pract Technol. 2022;18: 16–26. doi:10.2166/wpt.2022.155*

*12.	Katipoğlu OM, Sarıgöl M. Improving the accuracy of rainfall-runoff relationship estimation using signal processing techniques, bio-inspired swarm intelligence and artificial intelligence algorithms. Earth Sci Inform. 2023;16: 3125–3141. doi:10.1007/s12145-023-01081-w*

*13.	Katipoğlu OM, Yeşilyurt SN, Dalkılıç HY, Akar F. Application of empirical mode decomposition, particle swarm optimization, and support vector machine methods to predict stream flows. Environ Monit Assess. 2023;195: 1108. doi:10.1007/s10661-023-11700-0*

*14.	Hameed MM, Mohd Razali SF, Wan Mohtar WHM, Yaseen ZM. Examining optimized machine learning models for accurate multi-month drought forecasting: A representative case study in the USA. Environmental Science and Pollution Research. 2024. doi:10.1007/s11356-024-34500-6*

*15. Hameed MM, Masood A, Srivastava A, Abd Rahman N, Mohd Razali SF, Salem A, et al. Investigating a hybrid extreme learning machine coupled with Dingo Optimization Algorithm for modeling liquefaction triggering in sand-silt mixtures. Sci Rep. 2024;14: 10799. doi:10.1038/s41598-024-61059-6*

*15. Alomar MK, Khaleel F, Aljumaily MM, Masood A, Razali SFM, AlSaadi MA, et al. Data-driven models for atmospheric air temperature forecasting at a continental climate region. PLOS ONE. 2022;17: e0277079.*

*16.	Döll P, Abbasi M, Messager ML, Trautmann T, Lehner B, Lamouroux N. Streamflow Intermittence in Europe: Estimating High-Resolution Monthly Time Series by Downscaling of Simulated Runoff and Random Forest Modeling. Water Resources Research. 2024;60: e2023WR036900. doi:https://doi.org/10.1029/2023WR036900*

*17.	Zema DA, Parhizkar M, Plaza-Alvarez PA, Xu X, Lucas-Borja ME. Using random forest and multiple-regression models to predict changes in surface runoff and soil erosion after prescribed fire. Modeling Earth Systems and Environment. 2024;10: 1215–1228. doi:10.1007/s40808-023-01838-8*

5. The algorithms of machine learning models should be simplified, and the innovation of this paper should be highlighted. Readers are more concerned about the advantages of the deep learning model proposed in this study in forecasting.

*Reply: the algorithms have been simplified and summarized as per the reviewer’s recommendation. Please see page 13, and page 14 for further details.*

6. Line no 62, " Several studies [11–13]" the citation must be at the end of sentence.

*Reply: References are moved to the end of the sentence as suggested.*

7. Lin No 79, the referencing and citation must be according to the journal style. for instant, water table [28] and river stages (Choi et al., 2020), both type of citations are there. it should be check throughout the manuscript.

*Reply: The references have been checked and arranged according to the journal's style guidelines.*

8. Due to the extensive use of abbreviations, it is essential to include a list of acronyms for clarity and ease of understanding.

*Reply: The authors have now included a separate table for abbreviations, which has been added toward Appendix in the supplementary file.*

9. More datasets/no of stations should be provided to demonstrate the generalization ability and robustness of AI models.

*Reply: We have provided additional details and data to demonstrate the generalization capacity of the models, as suggested. Please refer to the Results section on page 26-27, lines 454 to 465, page 27-28, lines 481 to 489, and page 30, as well as Figure 8 and Table 5.*

10. What is new in this manuscript that was not there in the previous study? And what are the differences between this study and the old study? The limitations should be differentiated in discussion.

*Reply: We have improved our paper based on your suggestions:*

1- *We compared the performance of our models with other models developed in the literature and found that our model demonstrates superior performance. Please see the discussion section on pages 35-36 (lines 576- 599).*
2- *In our study, we used “turning point analysis” to evaluate the performance of the models, which has not been done before in runoff modeling. We explained this in the discussion section to highlight the novelty and differences between this study and previous research. Consequently, the discussion section has been enhanced as per your suggestion."*

11. Future study must be stated.

*Reply: Recommendations for future research have been included in the conclusion section. Please see the last paragraph on pages 40 and 41.*

Reviewer #3: The article titled

"Forecasting Monthly Runoff in a Glacierized Catchment: A Comparison of Extreme Gradient Boosting (XGBoost) and Deep Learning Models" evaluates the effectiveness of XGBoost, LSTM, and Random Forest RF models in forecasting monthly runoff in the Lotschental catchment, Switzerland. This comparative analysis is beneficial for identifying the best model for monthly runoff forecasting under complex hydrological conditions, especially in glacierized catchments.

The article is interesting, but the following issues should be addressed.

Specific Comments and Suggestions:

1. Consider adding in Abstract a sentence highlighting the novelty of the study, such as the use of turning point analysis or the comparison of multiple machine learning models in this specific context.

*Reply: We have highlighted the contributions of the research in the Abstract section, emphasizing the comparison between deep learning and ensemble models, the application of turning point analysis, and the modeling of monthly runoff in glacierized catchments affected by climate change.*

2. In Introduction the literature review could be further strengthened by discussing recent advancements in machine learning and their potential applications in hydrological modeling.

*Reply: Thank you for your insightful suggestion. We have enhanced the literature review in the Introduction and have incorporated the most recent references which highlight recent advancements in machine learning and their emerging applications in hydrological modeling.*

3. The research questions could be explicitly stated at the end of the Introduction to provide a clear focus for the study.
*Reply:* *We have incorporated the research question as suggested. Please see pages 8 and 9 (lines, 178 to 185).*

4. Data Sources: Clearly indicate the specific sources of meteorological and hydrometric data used in the study.
*Reply: In this study, we utilized runoff time series data. The data used are provided in the Appendix (Table S1), and we highlighted the reference for the data in the main paper (Section of Case Study Location, page 11).*

5. Additional questions for consideration In the "Case Study Location" section:
- have there been any significant changes in land cover or vegetation patterns within the catchment in recent decades?
- are there any known geological faults or other factors that could influence the stability of the slopes and the risk of landslides?
- how have past flooding events affected the local communities and infrastructure?

*Reply: We have now included the requested information on the Canton of BERN, which encompasses both our study area and additional regions. This was necessary due to the lack of specific studies available for our exact study area. Please see pages 9.*

6. Limited data temporal scope.
Although the study spans nearly two decades (2002-2021), integrating longer historical data could provide a more comprehensive understanding of long-term trends and improve the models' generalization capacity. Then the models could capture rare but critical climate events that affect runoff Why weren't earlier data and longer measurement series used?

*Reply: We appreciate the reviewer’s insightful suggestion regarding the inclusion of longer historical data. While we acknowledge that extending the dataset further back in time could enhance the understanding of long-term trends and capture rare climate events, our analysis was constrained by the availability and consistency of high-quality observational data. Earlier records often suffer from missing values, inconsistent measurement methodologies, or limited spatial coverage, which could introduce biases and reduce the reliability of the models. By focusing on the 2002–2021 period, we ensured the use of robust, consistent, and reliable data to produce defensible results while addressing recent trends and their implications. Future work could explore extending the dataset if reliable historical records become accessible, allowing for an improved assessment of rare climate impacts on runoff.*

7. Consider adding a more detailed explanation of the LSTM architecture.

*Reply: We have provided more* detailed explanation of the LSTM architecture. Please see page 14, and figure 3b.

8. Discuss the process of hyperparameter tuning for each model, as this can significantly impact performance.
*We have discussed the selection of hyperparameters for each model, as suggested, and provided a table detailing the hyperparameters used in this research. Please refer to the Results and Discussion section on page 25, and Table S2 in the Appendix, supplementary file.*

9. How did the choice of input lags affect the performance of the models?
*Reply: In this study, we have used three input scenarios. We have enhanced the discussion section to address how the input lags affect the performance of the models, as per your suggestion. Please see pages 38 and 39, lines 628 to 649; for further details.*

10. Were any other machine learning models considered for comparison?
*Reply: we have compared the performance of our models with several machine learning models which developed recently in previous research. The models which developed in prior research included for example but not limited, hybrid models such as extreme learning machine with grey wolf optimization (*ELM-GWO), Emotional Artificial Neural Network, support vector regression, and others. These comparisons are provided in the discussion section, pages 35, and 36, lines 576 to 599.

11. Were there any significant differences in model performance during different seasons or hydrological conditions?
*Reply:* *We have incorporated a discussion and detailed analysis of the performance of each model during different months and seasons, as suggested. Please refer to pages 26, and 27.*

12. How did the models perform in forecasting extreme events, such as floods or droughts?
*Reply:* *We have enhanced the Results section of this paper by discussing how the applied models simulate both the highest and lowest runoff values. This provides a better understanding of the models' performance during different hydrological events, including floods and droughts. Please see pages 27, and 28.*

13. While the study compares three individual models, it doesn’t explore hybrid modeling approaches. Combining the strengths of XGBoost and LSTM, for instance, could yield a model that balances the interpretability and robustness of XGBoost with the sequential learning advantages of LSTM. The authors should refer to the issue of potential for hybrid model.

*Reply: We compared our models with hybrid models developed in previous studies and found that our model performed better. This may be attributed to the hybrid models requiring more input data. We also acknowledge that the comments highlight one of the limitations of this study. Furthermore, we faced data limitations, as only time series runoff data was available. Developing advanced hybrid models requires multiple climate parameters to achieve significant improvements over classical models. Therefore, we recommend that future studies incorporate satellite data and develop advanced hybrid models for comparison with LSTM and XGBoost models.*

14. Although LSTM is a robust model for time series data, other advanced deep learning architectures, could further improve forecasting accuracy by capturing long-term dependencies more effectively. The authors should address this problem.

*Reply: Thank you for your valuable feedback. While we recognize that advanced deep learning architectures, such as Transformers or attention-based models, may offer potential advantages in certain contexts, we specifically selected the LSTM model for its demonstrated effectiveness in time series forecasting, particularly for runoff data. LSTM's ability to capture both short-term and long-term dependencies through its gating mechanisms makes it particularly suitable for hydrological data, where understanding the relationships between past inputs and future outputs is critical. Moreover, LSTM is considered an improved version of the RNN algorithm, which is prone to vanishing gradient issues* (Hameed et al., 2024; Le and Zuidema, 2016; Noh, 2021)*, making LSTM a more robust choice for time series data modeling . Additionally, LSTM has been widely acknowledged in the hydrology domain for its reliability in handling sequential data and its ability to model complex temporal patterns, which are essential for accurate runoff prediction.*

*Hameed, M.M., Mohd Razali, S.F., Wan Mohtar, W.H.M., Ahmad Alsaydalani, M.O., Yaseen, Z.M., 2024. Deep learning versus hybrid regularized extreme learning machine for multi-month drought forecasting: A comparative study and trend analysis in tropical region. Heliyon 10, e22942. https://doi.org/https://doi.org/10.1016/j.heliyon.2023.e22942*

*Le, P., Zuidema, W., 2016. Quantifying the vanishing gradient and long distance dependency problem in recursive neural networks and recursive LSTMs. arXiv Prepr. arXiv1603.00423.*

*Noh, S.-H., 2021. Analysis of gradient vanishing of RNNs and performance comparison. Information 12, 442.*

15. The sensitivity of the AETP metric should be assessed to different thresholds for defining turning points. This can help to evaluate the robustness of the results.
*Reply: We have provided the threshold for the AETP metric to effectively evaluate the robustness of the results. For further details, please refer to page 23, lines (386-390).*

16. Including climate projections could significantly enhance the model's utility for long-term water resource planning in glacierized regions that are highly sensitive to global warming.
*Reply: Thank you for your helpful comment. Unfortunately, we did not have access to climate data for this study, which could have provided additional context and potentially improved the model's performance. We recognize the value of incorporating climate variables such as precipitation, temperature, and humidity, as they can play a significant role in runoff prediction. In future work, we suggest for researchers to include climate data to enhance the accuracy and robustness of our model, and we believe this will offer more comprehensive insights into the factors influencing runoff*

17. Are there any specific regions or time periods where one model consistently outperforms the others?
*Reply: We have improved the results based on your constructive comments. Our findings indicate that XGBoost consistently outperforms the other models across all seasons, particularly from April to June. For more details, please see page 26, and 27, lines (454-465)*

18. To improve the "Discussion" section, it would be necessary to specify"
- how do the findings of this study compare to other research on runoff forecasting in glacierized catchments located in different regions?
- are there any potential biases or uncertainties associated with the use of historical data for model training and validation?
- could the XGBoost model be adapted or improved to forecast runoff at different time scales (e.g., daily, weekly)?

Incorporating longer data records, exploring hybrid models, and integrating climate change scenarios could further enhance the model's applicability in hydrological forecasting.

*Reply:*

- *As per your suggestion, the discussion section has been improved by comparing our results with findings from other researchers on runoff forecasting in glacierized catchments located in different regions. Please refer to the page 36, lines 587 to 599*
- *We have enhanced the discussion on data bias as suggested. Please see pages 37 and 38, lines 615 to 627.*
- *We have improved the discussion section by addressing the potential of the XGBoost model to forecast runoff at different scales, such as daily and monthly, as suggested.*
- *This paper is limited to a specific runoff dataset covering the period from 2002 to 2021. Therefore, we propose that future studies incorporate longer data records and climate data, which can be obtained from observed stations or satellite data. Acquiring larger datasets could help enhance hybrid models and allow for exploration of how climate change affects runoff.*
